# Driving Performance in Schizophrenia: The Role of Neurocognitive Correlates—A Systematic Review

**DOI:** 10.3390/brainsci15101094

**Published:** 2025-10-10

**Authors:** Georgia Karakitsiou, Spyridon Plakias, Aikaterini Arvaniti, Magdalini Katsikidou, Katerina Kedraka, Maria Samakouri

**Affiliations:** 1 Department of Psychiatry Medical School, Democritus University of Thrace, 68100 Alexandroupolis, Greece or aarvanit@med.duth.gr (A.A.); mkatsiki@med.duth.gr (M.K.); msamakou@med.duth.gr (M.S.); 2 Department of Physical Education and Sport Science, University of Thessaly, 38221 Trikala, Greece; spyros_plakias@yahoo.gr; 3 Department of Molecular Biology and Genetics, Democritus University of Thrace, 68100 Alexandroupolis, Greece; kkedraka@mbg.duth.gr

**Keywords:** driving performance, fitness to drive, schizophrenia, neuropsychology, cognitive impairment, cognitive function

## Abstract

**Background/Objectives**: Schizophrenia is associated with cognitive deficits that may compromise everyday functioning, including driving. This review systematically examined recent original research (2015–2025) on driving performance in individuals with schizophrenia with a focus on neuropsychological factors, applying a narrative synthesis given the heterogeneity of designs and outcomes, while no quantitative meta-analysis was feasible. **Methods**: Following the PRISMA (Preferred Reporting Items for Systematic Reviews and Meta-Analyses) guidelines, a structured search of PubMed and Scopus was conducted on 4 May 2025. The inclusion criteria were original studies involving individuals diagnosed with schizophrenia, published between 2015 and 2025. Studies on animals, other psychiatric or neurological conditions, and healthy populations were also excluded. Critical appraisal was performed using the Joanna Briggs Institute (JBI) tools. Extracted data included sample demographics, cognitive deficits, neuropsychological assessments, brain imaging, and the main findings. A narrative synthesis was then performed. **Results**: Six high-quality studies met the inclusion criteria. Findings were grouped into three categories: (1) driving behavior: fitness to drive varied widely across individuals, (2) cognitive deficits and brain activity: poorer driving-related performance was consistently associated with specific impairments in cognition and brain structure, and (3) medication effects: individuals taking certain atypical antipsychotics demonstrated better driving performance compared to those on other types of medication, while extrapyramidal symptoms negatively influenced driving fitness. **Conclusions**: Driving in schizophrenia is shaped by cognitive, clinical, and pharmacological factors. These findings highlight the clinical relevance of individualized evaluations, integration into personalized care and targeted rehabilitation to promote driving autonomy and community inclusion. This area remains under-researched, as only six studies met the inclusion criteria, which restricts the robustness and generalizability of the conclusions. Funding: This review received no funding from any external sources. Registration: The review protocol was submitted to PROSPERO (International Prospective Register of Systematic Reviews) under registration number CRD420251060580.

## 1. Introduction

Schizophrenia is a mental disorder in which, according to the DSM-5 criteria (Diagnostic and Statistical Manual of Mental Disorders, Fifth Edition) [1], patients may exhibit delusions, hallucinations, disorganized speech, grossly, disorganized or catatonic behavior, and negative symptoms, such as diminished emotional expression or avolition. For a diagnosis of schizophrenia, continuous signs of the disorder must be present for at least six months, with at least one month of active-phase symptoms [2,3,4]. In addition, for a significant portion of this time, one or more major areas of daily functioning such as work, interpersonal relationships, or self-care must be markedly impaired compared to the level prior to the onset of this mental disorder [2,5]. Schizophrenia is associated with very high direct healthcare costs for patient care as well as significant indirect costs due to productivity loss [6]. Due to the severity of symptoms, patients with schizophrenia appear to face a wide range of difficulties and widespread disability across multiple domains of everyday functioning [7].

Some of the commonly reported challenges for individuals living with schizophrenia in the literature are associated with cognitive and emotional functions, interpersonal relationships and employment [8]. Importantly, their actual performance in everyday life often falls below their potential functional capacity, a discrepancy that highlights the clinical relevance of evaluating real-world functioning [9,10]. Recent evidence further emphasizes that functional outcomes and quality of life are strongly influenced not only by symptoms but also by the effects of treatment. For example, Sampogna, et al. [11] systematically analyzed how antipsychotic treatment affects quality of life in schizophrenia, providing a robust framework to contextualize the link between cognitive performance and daily functioning.

Due to the severe difficulties observed in patients with schizophrenia, high levels of self-stigma have been identified. This means that patients themselves internalize stigma related to their mental disorder, while their levels of self-stigma are significantly influenced by socio-economic factors, the absence of a partner, the use of high doses of medication and low functioning [12]. Valery and Prouteau [13] also indicated that schizophrenia is one of the most stigmatized mental disorders by mental health professionals. Conclusively, it seems that individuals with schizophrenia represent a distinct population group who, due to the severity of the disorder, face numerous challenges and difficulties. These difficulties significantly impair their ability to perform daily activities, thus hindering their smooth integration into society and putting them at risk of marginalization and social isolation.

Regarding the issue of social isolation, findings indicate that involvement in driving activities significantly enhances social integration and an individual’s quality of life [14]. As a highly complex task requiring the integration of multiple cognitive functions, it is particularly vulnerable to impairment. Studies in both healthy populations and those with mild cognitive impairment (MCI) confirm that reduced cognitive ability predicts poorer driving outcomes [15,16,17]. Thus, neuropsychological assessments are a critical component of multidisciplinary evaluations of driving ability [18].

According to Bascom and Christensen [19] the issue of transportation for individuals with disabilities, is considered to be of great importance, as it was found that limited access to transportation significantly hindered their social life, according to self-reports. Their study revealed that individuals with disabilities use private vehicles less frequently than public transportation, while the severity of the disability influences their sense of exclusion from society. Moreover, during a review of the relevant literature, it was found that the issue of driving ability in patients with schizophrenia has only been minimally reviewed. More specifically, we identified a systematic review by Unsworth, et al. [20], in which driving ability was evaluated across various mental health disorders and was not limited exclusively to patients with schizophrenia. The disorders examined included depression, anxiety, personality, and obsessive–compulsive disorder. Additionally, Fernandes, et al. [21] addressed the issue of driving in schizophrenia; however, this systematic review relied on research from previous decades, underscoring the need for an updated investigation into this topic. Of the final studies included in their systematic review, seven in total, only two were conducted in 2015, and none were published more recently. The remaining five studies are considerably older, leaving a significant gap in the international literature and underscoring the need to update this area of inquiry and re-evaluate it considering more current empirical evidence.

Given the significance of driving for autonomy and social integration, and its strong association with cognitive and neuropsychological deficits in schizophrenia, this review aimed to systematically examine and synthesize the recent existing research (2015–2025) on driving performance in individuals with schizophrenia. It further aimed to identify research gaps and propose new directions for future investigations, emphasizing the role of neuropsychological factors that are related to fitness to drive in schizophrenia.

## 2. Materials and Methods

### 2.1. Reporting

The review was conducted according to the PRISMA guidelines [22,23] (see Supplementary File S1 for the PRISMA checklist) and was registered with PROSPERO (ID: CRD420251060580). It did not require ethical approval, as it relies exclusively on data from studies that had already received ethics approval upon their original publication.

At all stages of the review disagreements between the two independent reviewers were resolved through discussion. When consensus could not be achieved, a third independent author, not involved in the initial assessment, was consulted to arbitrate. This procedure was pre-specified in the PROSPERO protocol to ensure methodological transparency and consistency.

### 2.2. Search

Two investigators (G.K. and S.P.) conducted a literature search of two databases (Medline and Scopus) to trace all relevant studies until 4 May 2025. Any disagreement regarding the screening or selection process was further discussed with a third investigator (M.S.) until consensus was reached.

The search strategy applied to the databases was as follows:

- Scopus

TITLE-ABS-KEY ((“Neuropsychological exponents” OR “cognitive deficits” OR “cognitive disorders” OR “cognitive functions”) AND (driv* OR “driving fitness”) AND schizophrenia) AND PUBYEAR > 2014 AND PUBYEAR < 2026 AND (LIMIT-TO (DOCTYPE, “ar”)) AND (LIMIT-TO (LANGUAGE, “English”)) AND (LIMIT-TO (EXACTKEYWORD, “Human”)).

- Pubmed

((((neuropsychological exponents) OR (cognitive deficits) OR (cognitive disorders) OR (cognitive functions)) AND (driv*) OR (“driving fitness”)) AND (schizophrenia))

Filters applied: in the last 10 years, English, Humans

PubMed and Scopus were chosen as the core databases for this systematic review due to their broad coverage of biomedical and psychological literature, including psychiatry, neuroscience and cognitive sciences. These databases are widely recommended and frequently used in systematic reviews in the mental health field, ensuring comprehensive retrieval of relevant studies. Furthermore, to minimize the likelihood of missing eligible studies, we further applied backward reference list checking and forward citation tracking of the included studies and relevant reviews, in order to minimize the risk of omitting potentially eligible articles. This combined approach was deemed sufficient to capture the existing body of evidence on driving performance in schizophrenia.

### 2.3. Selection Criteria

We included only original full-text research articles published in English and studies published within the last ten years, specifically between 2015 and 2025. We chose to include only studies conducted within the last decade, as our aim was to update the research data on this topic and to explore more recent investigations, helping to supplement and bring the research data up to date, thus complementing the systematic review by Fernandes, et al. [21] who examined the same issue by including earlier studies. Secondary analyses, reviews, guidelines, meeting summaries, comments, unpublished abstracts and studies conducted with animals were excluded. The sample characteristics were strictly limited to populations diagnosed with schizophrenia, and any other population group was excluded. Inclusion exclusion criteria are presented in Table 1.

### 2.4. Data Extraction and Analysis

Two reviewers (G.K. and S.P.) independently worked for the data extraction and analysis of each study. Any discrepancies were resolved through discussion, and when a consensus could not be reached, a third author (K.K.) was consulted to make the final decision.

Data were extracted using a predefined form created in Microsoft Excel. We recorded the authors, year of publication, article title, study origin, patient characteristics (total number, number by gender and age), whether the study included a control group or not, and in cases where a control group was included, the participants’ characteristics (total number, number by gender and age). Additionally, the identified cognitive deficits, neuropsychological tests used to assess these deficits, and whether and how driving ability was measured were recorded. We also noted whether brain scans were used in the included studies as well as the reported neuroanatomical findings and we documented whether any type of intervention or rehabilitation was performed with the participants in the included studies, along with the key findings from each study. Information on pharmacological treatment was not extracted as a predefined variable in our review. However, medication use and side effects were reported narratively when they appeared in the main findings of the included studies. In addition, we examined whether the included studies reported the use of complementary investigations to exclude organic causes of psychosis or to examine the presence of secondary schizophrenia. Results of individual studies and the synthesis were presented in tables and figures summarizing demographics, assessment methods, cognitive findings, and driving performance.

No statistical analysis or meta-analysis was performed because of the high heterogeneity observed among the studies. Thus, the data were only descriptively analyzed. No sensitivity analyses were conducted due to the descriptive nature of the synthesis, and no specific statistical methods were applied to assess risk of bias due to missing results. A narrative synthesis was chosen due to the substantial heterogeneity in study designs, sample sizes, outcome measures, and neuropsychological tests, which precluded meaningful statistical pooling. Given the small number of eligible studies (*n* = 6) and their methodological diversity, even exploratory subgroup or sensitivity analyses were not feasible.

### 2.5. Quality Assessment

Two reviewers (G.K. and A.A.) independently assessed the quality and validity of each study. Any discrepancies were resolved through discussion, and when a consensus could not be reached, a third author (M.K.) was consulted to make the final decision.

An amendment was made to the planned methodology regarding the assessment tools. Although we initially specified at the PROSPERO protocol that the Newcastle-Ottawa Scale (NOS) would be used to assess study quality in this systematic review, during the final selection process it became apparent that all studies that met the eligibility criteria were identified as Analytical Cross-Sectional designs. While the Newcastle–Ottawa Scale (NOS) is widely used to assess the quality and risk of bias in other types of studies, it has several limitations, particularly when applied to cross-sectional designs [24].

Consequently, the Joanna Briggs Institute (JBI) critical appraisal tools were employed as they provide design-specific checklists appropriate for a wide range of study types [25] among which a specialized tool for the appraisal of analytical cross-sectional studies [26]. These appraisal tools have also been widely used in other systematic reviews to evaluate credibility and adaptability across included studies [27,28,29].

### 2.6. Assessment of Certainty in the Body of Evidence

Given the high heterogeneity in study design, outcome measures, and methodological approaches among the included studies, a formal quantitative tool such as GRADE (Grading of Recommendations Assessment, Development and Evaluation) was not applied. Instead, a narrative assessment of certainty was conducted by considering several key dimensions across the body of evidence. These included the overall methodological quality of the studies (as determined by the JBI appraisal tools), consistency of findings across studies, directness of the evidence with respect to the review question and the presence of clearly reported outcomes.

## 3. Results

### 3.1. Database Searches

**Figure 1:**
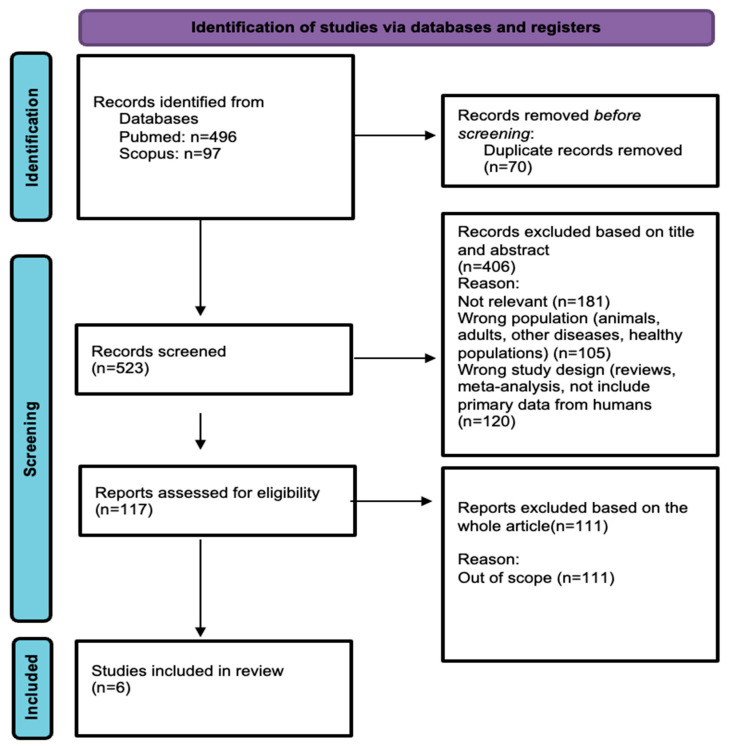
Flowchart of the literature search according to the PRISMA guidelines.

A total of 593 records were retrieved from the database search (Scopus, 97; PubMed, 496). After removing duplicates, 523 unique studies remained for screening. Titles and abstracts were reviewed for relevance according to predefined inclusion and exclusion criteria. Specifically, 181 studies were excluded because they were irrelevant, 105 were excluded because they involved different populations (exempli gratia-e.g., animals, healthy individuals, or individuals with other medical or psychiatric conditions), and 120 were excluded due to inappropriate study design, including reviews, meta-analyses, or studies that did not involve original primary human data. Subsequently, 117 full-text articles were assessed for their eligibility. Of these, 111 were deemed out of scope. Ultimately, six articles met all the eligibility criteria and were included in the review. A flowchart of the literature search process is presented in Figure 1 [22].

### 3.2. Quality Assessment of Included Studies

All included articles were classified as analytical cross-sectional studies (*n* = 6). As evidenced by the review in Table 2, the totality of the studies included was of high methodological quality, and they met most of the criteria for inclusion in the systematic review. A detailed evaluation of the articles is shown in Table 2.

### 3.3. Data Extraction

#### 3.3.1. Study Design and Characteristics

In this systematic review, we included six original peer-reviewed studies that explored driving performance in individuals with schizophrenia. All the included studies employed quantitative methodologies with clearly defined patient populations and standardized cognitive assessment procedures. The review adhered to structured eligibility criteria, focusing on the inclusion of clinical schizophrenia populations, use of validated neuropsychological testing tools, and adequate reporting of demographic and methodological characteristics. Due to the high heterogeneity of study designs, populations, and outcome measures, no statistical synthesis, meta-analysis, or sensitivity analyses were conducted. Study characteristics and potential sources of bias were summarized narratively, and possible causes of heterogeneity were considered qualitatively and the results are presented in detail below.

**Figure 2:**
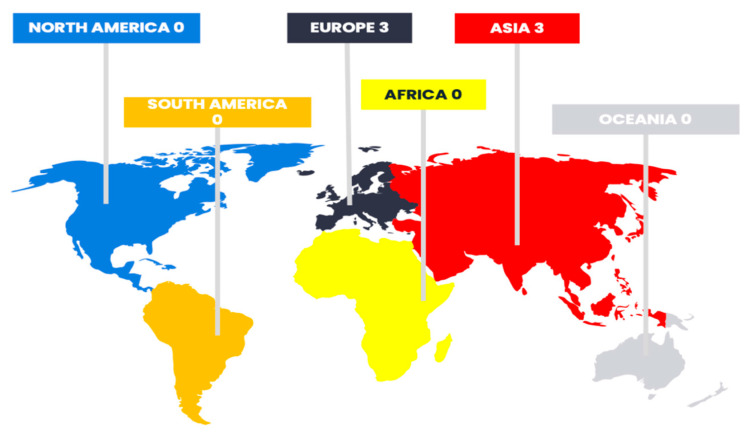
Geographic Distribution of Studies Included in the Systematic Review.

All the studies included in this review originated exclusively from Europe and Asia, with three studies conducted in each region, as illustrated in detail in Figure 2. More specifically, all studies from Europe were conducted in Germany, while among Asian studies, two originated from Japan and one from South Korea. Strikingly, no eligible studies were identified from other continents such as North America, South America, Africa, or Oceania. This highlights a significant geographical gap in the existing body of research and suggests that the topic remains markedly under-researched in large parts of the world, limiting the global generalizability and representativeness of the current evidence.

The sample size varied moderately across studies, ranging from a minimum of 20 participants to a maximum of 150 participants. No studies with large sample sizes were identified. All studies included a mixed-gender patient population, with the age range of patients across the included studies spanned from 29.9 to 46.3 years. Two studies incorporated healthy control groups, enabling a comparative analysis of driving ability. However, four studies focused exclusively on patient groups. Table 3 provides a detailed presentation of the findings. None of the six included studies reported the prevalence of patients undergoing complementary diagnostic investigations such as brain MRI, EEG, lumbar puncture, bloodwork or urine toxicology for the exclusion of organic causes. Stratification by primary versus secondary schizophrenia was not feasible; therefore, the included samples can be considered as representing primary schizophrenia. Lastly, participants in the included studies were predominantly clinically stable patients with mild to moderate symptomatology, while illness duration and functional status were not systematically reported across all studies.

#### 3.3.2. Assessment of Driving Ability

Among the six studies included in this review, only one employed an on-road driving test [31], offering the highest level of ecological validity for assessing real-world driving performance. One study assessed driving ability using a self-reported questionnaire [34], relying on participants’ retrospective accounts of their driving behavior. The remaining four studies utilized off-road driving evaluations, primarily simulator-based or computer-based assessments, which allowed for a standardized and controlled examination of driving-related performance in individuals with schizophrenia [14,32,33,35].

Off-road evaluations were based on computerized testing platforms designed to simulate driving-related tasks. For instance, the Vienna Test System and CPAD (Certificate for Professional Aptitude in Driving) provide detailed cognitive profiles by measuring depth perception, reaction time, divided attention, and other skills critical to driving. These tools allow for standardized comparisons, although they lack a real-world driving context. Although off-road methods enhance consistency and safety and closely simulate real-world conditions, the limited use of real-world driving assessments highlights a gap in ecologically valid measurement approaches in this field.

#### 3.3.3. Assessment of Cognitive Deficits

Findings from the six studies included in this review showed that cognitive deficits were consistently linked to driving performance in individuals with schizophrenia. The cognitive deficits most frequently identified involved attention, memory, visuospatial abilities, processing speed, executive function, inhibition, planning, and hazard perception. It should be noted that the most reported finding across the included studies was related to attention and memory deficits. The aforementioned domains are considered particularly relevant for safe driving as they influence one’s ability to anticipate events, make decisions, and respond appropriately to sudden changes in the driving environment.

The neuropsychological assessment of cognitive functioning was performed using a range of standardized and validated instruments. The most commonly used test was the Trail Making Test A and B (TMT-A/B), which serves as a highly practical tool for evaluating neuropsychological skills [36]. Other clinical and neuropsychological assessment tools were also used, such as the Continuous Performance Test (CPT), Zoo Map Test (ZMT), subtests of the Wechsler Memory Scale (WMS), and Positive and Negative Syndrome Scale (PANSS). In addition to these core instruments, several other tests were employed across studies to assess various cognitive abilities and aspects of daily functioning, such as the Go/No-Go task, Digit Span, Useful Field of View (UFOV), and Global Assessment of Functioning (GAF). Some studies employed computerized test batteries specifically designed to evaluate driving-related cognition, such as the Vienna Test System and the Computerized Psychometric Assessment for Driving (CPAD), which assesses reaction time, depth perception, attention, inhibition and executive function.

#### 3.3.4. Neuroimaging

It is noteworthy that only one study [33], out of the 6 included, incorporated neuroimaging techniques. This study utilized functional near-infrared spectroscopy (fNIRS) to examine brain activation patterns during cognitive tasks relevant to driving. The findings revealed reduced cerebral blood flow in the prefrontal cortex, suggesting functional hypoactivation in brain regions that are critical for safe driving. No neuroanatomical findings were reported in the remaining studies, as neuroimaging was not employed elsewhere in the reviewed literature.

#### 3.3.5. Interventions and Treatment Variables

None of the six included studies included in this review implemented any form of intervention or rehabilitation targeting cognitive or driving-related functioning. This indicates a significant gap in the literature regarding evidence-based strategies to enhance driving competence in individuals with schizophrenia. Future research should consider incorporating and evaluating structured interventions aimed at mitigating the cognitive deficits that affect driving performance.

Although pharmacological treatment variables were not extracted as a primary outcome in this review, several studies explicitly reported medication type or side effects as part of their main findings. Specifically, side effects were assessed with standardized instruments such as the Drug-Induced Extrapyramidal Symptoms Scale (DIEPSS) and the Modified Simpson Angus Scale (MSAS). Moreover, medication use was described either in terms of olanzapine-equivalent doses or by comparing the effects of specific antipsychotic monotherapies (e.g., aripiprazole, paliperidone, haloperidol, risperidone). These details, as reported by the original studies, are summarized narratively to provide a clearer account of the pharmacological variables that may have influenced driving outcomes.

#### 3.3.6. Main Findings

Across the six included studies, the main findings could be divided into three primary categories. The first category includes findings related directly to the driving behavior of individuals with schizophrenia. The second category encompasses findings concerning core cognitive deficits and findings related to brain activity that are associated with driving ability in patients with schizophrenia. Finally, the third category includes findings related to the impact of medication or its side effects on the driving ability of patients with schizophrenia.

The first category focuses on the driving behavior of individuals with schizophrenia. It was demonstrated that people with schizophrenia were generally found to drive more slowly and to commit fewer speeding or distraction-related violations compared to healthy individuals. However, driving ability varied significantly within this population, with some individuals fully or partially fitting to drive, whereas others did not. Driving performance was influenced not only by cognitive abilities but also by factors such as driving experience, depressive symptoms, history of psychiatric hospitalization and substance-related offenses. Interestingly, despite cognitive deficits, patients did not show more collisions or delayed reactions in critical situations, and even demonstrated better lane control. Nonetheless, slower driving often hinders other drivers, particularly when merging.

The second category addresses cognitive deficits and brain-related findings associated with driving ability. Impairments in attention, working memory, executive functioning and processing speed were strongly linked to suboptimal driving behaviors. Visual memory and general functional capacity were also found to affect steering control and overall driving fitness. Neurophysiological data suggested that reduced frontal lobe activity and slower visual processing in people with schizophrenia contribute to delayed reactions, particularly during braking. These findings underscore the importance of evaluating cognitive and neural functioning when assessing driving competence in this population.

The third category examines the role of medication and its side effects. Motor side effects, especially extrapyramidal symptoms, were found to negatively impact driving safety, often resulting in sudden braking or reduced driving performance. Age and higher doses of antipsychotic medication were also associated with poorer driving ability. Importantly, the type of medication appeared to matter, as patients taking certain atypical antipsychotics demonstrated better cognitive performance on tasks relevant to driving. These findings highlight the necessity of monitoring both side effects and medication regimens when evaluating or supporting driving ability in patients with schizophrenia. Table 4 provides a detailed presentation of the aforementioned findings.

For clarity and ease of comparison, we synthesized the main findings across studies in a summary table. Table 5 presents the cognitive domains most frequently reported in association with driving performance in people with schizophrenia, highlighting their specific impact on driving behavior and safety.

#### 3.3.7. Assessment of Certainty in the Body of Evidence

Overall, confidence in the available evidence was evaluated as moderate regarding the association of various factors, such as cognitive impairment, brain dysfunction, and pharmacological effects with driving ability in individuals with schizophrenia. This evaluation was based on the consistency of findings across studies, despite the heterogeneity observed in the assessment tools and population characteristics.

However, confidence in the evidence related to real-world driving performance was very limited/low, as only one of the six included studies [31] utilized an on-road assessment with high ecological validity. Most studies relied on off-road evaluations through simulators or computer-based tasks, which, although standardized and controlled, fail to capture the complexity and unpredictability of real-world driving environments. Consequently, further research using ecologically valid and on-road assessments is required to strengthen the certainty of the conclusions of this systematic review. This limitation substantially weakens the robustness and generalizability of the review’s conclusions, underscoring the urgent need for more ecologically valid, on-road studies.

## 4. Discussion

### 4.1. Overview

In summary, this systematic review included six original peer-reviewed studies that explored driving performance in individuals with schizophrenia. We aimed to identify research gaps and propose new directions for future investigations, emphasizing the role of neuropsychological factors related to fitness to drive in schizophrenia. The overarching goal was to synthesize evidence linking the neurocognitive profile of schizophrenia to functional driving performance, highlighting the potential risks and limitations faced by this population on this particular issue. Regarding the quality assessment of the studies included, their totality was of a high methodological quality.

The main findings identified according to the major category are discussed below Each section of the following discussion is divided into two parts: first, we summarize the findings from the six included studies, and second, we contextualize them within the broader supporting literature.

### 4.2. Driving Behavior of Individuals with Schizophrenia

#### 4.2.1. Findings from the Six Included Studies

The first category, encompassing findings from the six included studies related to the driving ability of individuals with schizophrenia, revealed inconsistencies, indicating that a generalized assessment of driving ability in this population may not be appropriate. Some studies found that individuals with schizophrenia exhibit safe driving practices. For example, Okada, et al. [31] observed that participants with schizophrenia drove at lower speeds, committed fewer violations related to distraction (e.g., mobile phone use), and showed no increase in collisions or delayed reactions, even demonstrated better lane control than healthy individuals. However, they drove significantly slower and caused more hindrance to rear vehicles while merging on the motorway [35]. This suggests a defensive style, possibly compensating for cognitive limitations. It should also be noted that findings suggesting potential strengths in driving performance (e.g., cautious style, better lane control) derive from very few studies, and therefore should be interpreted with caution and not generalized across the wider population.

Other studies documented difficulties. In particular, Steinert, et al. [34] revealed that 64% of people with schizophrenia held a driving license, but only 32% of them had driven in the past year and 24.7% had experienced license withdrawal. Overall, patients with schizophrenia drive significantly less compared to the general population. Systemic barriers, such as hospitalizations and substance-related offenses, also reduced driving frequency. The hypothesis that external factors contribute to the reduced driving activity of individuals with schizophrenia is further supported by the study of Okada, et al. [33], who found steering control was influenced by cognition, driving experience, and depressive symptoms. Limited planning, less experience and slower braking increased collision risk. Overall, Biedermann, et al. [14], reported that 44% of clinically stable outpatients with chronic schizophrenia were competent to drive, 20% were partially competent and 36% were deemed incompetent.

#### 4.2.2. Supporting Evidence from Broader Literature

Beyond the six studies included in our systematic review, several additional studies, although not meeting our inclusion criteria, have also examined driving performance in schizophrenia or related psychotic disorders. These provide supporting context but were not part of the systematic synthesis. Some evidence indicated higher risk: For example, Unsworth, et al. [20] found that individuals with psychotic disorders exhibited higher rates of reckless and negligent driving and were more frequently involved in serious traffic accidents. Also, Germain, et al. [37] found that individuals with schizophrenia made significantly more errors and caused more collisions while driving and their speed was notably lower. Moreover, Wylie, et al. [38] demonstrated lower driving performance in individuals living with schizophrenia and Brunnauer, et al. [39] found that approximately 25% of hospitalized patients with schizophrenia, who are preparing for outpatient treatment, exhibit severe impairments in driving ability, particularly in skills related to concentration and vigilance. Brunnauer and Laux [40] indicated that a considerable proportion of patients with partially remitted schizophrenia should be regarded as unfit to drive even when undergoing stable treatment with atypical antipsychotics. Finally, Soyka, et al. [41] stated that an acute psychotic episode renders an individual unfit to drive and engagement in driving abilities may typically be reinstated following a one-year period of complete symptom remission.

The conflicting findings that have been identified, ranging from evidence that individuals with schizophrenia drive safely to reports of their engagement in dangerous driving behaviors, may be attributed to various factors, such as methodological differences (simulation versus real-world settings), variations in assessment tools, or differences in participant characteristics (symptom severity, phase, and treatment adherence). They may also reflect healthcare systems, cultural attitudes, access to psychoeducation, or even personality traits. Notably, earlier research from as far back as 1978 pointed out that variability in driving competence depends on personality traits [42]. Such findings emphasize the need to avoid generalization and support personalized assessments while investigating the issue of driving ability in schizophrenia.

An additional methodological consideration relates to ecological validity: only one study used on-road tests, while most relied on simulators. Although simulators ensure safety and standardization, they cannot fully reproduce the complexity, unpredictability, and social context of real-world driving, limiting generalization. Future work should integrate simulators with naturalistic methods.

Moreover, some evidence suggests that individuals with schizophrenia who continue to drive may represent a subgroup with higher functioning or greater support systems [43] and tend to have better mental health than those who do not drive [21]. Thus, the act of driving may indicate the relative wellness of some individuals. Nonetheless, the variability in findings across studies highlights that schizophrenia should not be viewed as a uniform predictor of poor driving outcome. For instance, patients with well-managed symptoms and good insight may compensate for cognitive deficits by adopting more cautious driving behavior, which could partially explain the lower average speed found in some studies. In conclusion, driving assessment should account for cognitive, clinical, and psychosocial variables.

### 4.3. Cognitive Deficits and Brain Activity Associated with Driving Ability in Patients with Schizophrenia

#### 4.3.1. Findings from the Six Included Studies

According to the six included studies, cognitive impairments (attention, executive dysfunction, visual memory, low GAF) were linked to driving violations [31,34]. Moreover, Okada, et al. [33] revealed that reduced frontal lobe activity and slower visual processing speed are linked to delayed braking responses in individuals with schizophrenia. Finally, Fuermaier, et al. [35] showed that cognitive impairments, especially in attention, planning and inhibition, were closely related to suboptimal driving behaviors. These studies show driving deficits stem from cognitive and neural dysfunctions, supporting neurocognitive assessments over diagnosis alone.

In a study that investigated the prefrontal activation during simulated driving in people with schizophrenia which was conducted by Okada, et al. [44], it was found that the brain mechanisms related to driving are likely similar in patients with schizophrenia compared to healthy controls, as no statistically significant differences were observed in performance across any of the tasks applied. Although this finding appears inconsistent with broader literature, it may be explained by the characteristics of the specific sample, which may have included individuals with relatively preserved cognitive functioning. Additionally, methodological factors, such as the type of assessment tool used or the possible lack of ecological validity, could have limited the study’s ability to reflect the actual challenges of real-world driving.

#### 4.3.2. Supporting Evidence from Broader Literature

In addition to these six studies, broader literature not included in the systematic review has reported similar patterns. Segmiller, et al. [45] showed 58% had psychomotor impairments, Pardeller, et al. [46] linked attention and concentration with driving ability and Lipskaya-Velikovsky, et al. [47] found that lower visual perception predicted reduced driving.

These findings reinforce the view that driving competence in individuals with schizophrenia is determined less by the diagnosis itself and more by a person’s cognitive functioning, which can vary significantly from one patient to another. Therefore, there is a need to assess individual differences in cognitive ability. Moreover, the importance of using specialized screening tools that evaluate specific cognitive domains relevant to driving, such as attention, visual processing and decision-making, is highlighted, rather than relying solely on the clinical diagnosis of schizophrenia.

The literature has identified a multitude of studies highlighting cognitive deficits and brain dysfunctions in patients with schizophrenia, which significantly impact an individual’s daily functioning. More constantly, Aguirre, et al. [48] demonstrated problems in processing speed of individuals with schizophrenia, Granato, et al. [49] reported executive deficits in more impaired patients and attentional and reasoning issues in higher-functioning patients, while Wu, et al. [50] identified significant cognitive deficits across all patient groups. Moreover, Maes, et al. [51] identified executive and memory deficits and Reddy, et al. [52] identified neurocognitive deficits. Similarly, Wang, et al. [53] reported cognitive dysfunction and impairments, while Saleh, et al. [54], associated this dysfunction with reduced reward sensitivity.

In addition, a plethora of other studies indicate that cognitive impairment and dysfunction in schizophrenia are related to brain structure and abnormalities [55,56,57,58,59,60,61,62]. Specifically, Brandt, et al. [63] identified differences in working memory, which were associated with fronto-parietal activation and fronto-temporal cortical thickness. Poorer cognitive performance, especially processing speed and executive functioning was linked to higher inflammation levels, especially in women [43], with lower hippocampal glutamate [64], network deficiency [65], alterations in tryptophan catabolism [66], reduced volumes in specific brain areas [67] and reduced P3a and P3b amplitudes [68]. Additionally, connectivity in right-hemisphere sensory regions was linked to poorer attention [69], hippocampal shape abnormalities was linked to verbal working memory [70] and white matter aging was associated with cognitive deficits in working memory and processing speed [71].

As indicated by the referenced literature, cognitive impairments in schizophrenia are often pronounced and span a wide range of domains, suggesting that even individuals with higher levels of functioning may experience cognitive difficulties that affect complex daily activities, such as driving. Moreover, cognitive deficits in schizophrenia are not only observable at the behavioral level but are also underpinned by measurable neurobiological dysfunctions. This has important implications, as biological markers may, in the future, contribute to objective risk assessments regarding driving ability in this specific population group.

### 4.4. Impact of Medication on the Driving Ability

#### 4.4.1. Findings from the Six Included Studies

The six included studies provided limited but important data on the role of medication and its side effects on the driving ability. As noted in the Results, pharmacological treatment variables were inconsistently reported across studies. While not systematically extracted, side effects and medication use were described narratively based on the original articles.

A recurring finding concerned the impact of typical antipsychotics (first-generation), particularly haloperidol. Sudden braking behavior was linked to extrapyramidal symptoms, suggesting an interplay between motor side effects and driving safety [31]. Moreover, driving fitness was negatively associated with age, higher olanzapine equivalent doses and the presence of extrapyramidal symptoms, with the severity of those symptoms emerging as the strongest predictor of reduced driving ability. Residual symptoms had weaker effects [14]. These findings suggest that careful monitoring of extrapyramidal symptoms and medication dosage is essential for evaluating the driving capability of individuals with schizophrenia. In this light, it seems that physical and neurological side effects may pose greater risks than core psychiatric symptoms. This is consistent with the broader evidence that first-generation (typical) agents, such as haloperidol, are more prone to induce motor impairment compared to second-generation (atypical) antipsychotics.

In contrast, atypical antipsychotics (second-generation) showed a more favorable profile in certain studies. Notably, Noh, et al. [32] found that individuals treated with aripiprazole or paliperidone demonstrated superior cognitive performance in driving-related tasks (depth perception, divided attention, digit span, and trail-making) compared to those treated with haloperidol or risperidone, suggesting that the type of antipsychotic medication may influence cognitive functioning and partly explain variability in outcomes. Such results underline the clinical relevance of distinguishing between typical and atypical antipsychotics, with the latter generally associated with fewer extrapyramidal side effects and a more favorable cognitive profile.

#### 4.4.2. Supporting Evidence from Broader Literature

Beyond the included studies, additional research, although outside our inclusion criteria, offers supporting insights into how different antipsychotics and side effects may influence driving ability. For example, Soyka, et al. [72] examined how different types of medication for the treatment of schizophrenia, affected psychomotor performance, which is considered highly important for safe driving. They found that patients treated with risperidone showed better performance compared to those treated with haloperidol. Clozapine and other atypicals improved attention, stress resilience, and concentration compared to typicals [73]. Other work found no clear differences among certain atypicals [40]. Risperidone patients also outperformed haloperidol in competence rates [74]. Finally, Pardeller, et al. [46] confirmed extrapyramidal symptoms impair driving. Although some studies highlight clear differences between typical and atypical antipsychotics, others report no statistically significant variations, suggesting that additional moderating factors may be involved, such as patient age, illness duration, comorbidities, or even genetic differences in drug metabolism. Nevertheless, the consistent pattern across multiple studies is that typical antipsychotics tend to impair driving performance through motor side effects, whereas atypical antipsychotics often present a safer cognitive and psychomotor profile. This distinction has practical implications, as clinicians may prioritize atypical agents when maintaining patients’ autonomy and driving safety is a treatment goal. This inconsistency highlights the need for standardized evaluation methods.

The literature also provides further research findings that highlight the effectiveness not only of certain medications, but also of therapeutic interventions aimed at improving specific cognitive abilities in individuals with schizophrenia. For example, it was highlighted that cognitive function and working memory may benefit from changes in clozapine levels and the way it is metabolized in the body [75]. Executive function and general cognition can be improved by Targeted Cognitive Training (TCT) [76,77,78], while general functioning may be supported by gains in working memory and processing speed [79]. In general, personalized approaches to schizophrenia care may help to predict better treatment outcomes [80]. Overall, treatment effects vary, so interventions must be individualized, moving beyond symptom control to functional driving skills.

### 4.5. Overall Perspective and Implications for Clinical Practice

The findings of this systematic review highlight a complex and multidimensional relationship between schizophrenia and driving ability, shaped by cognitive, behavioral, neurobiological and pharmacological factors. Diagnosis alone is not a reliable predictor as driving ability varies widely. Some individuals compensate for cognitive difficulties by adopting more cautious driving strategies, while others are at greater risk of engaging in unsafe driving behaviors. Notably, driving is shaped by both internal (cognition, medication) and external (experience, hospitalizations, support) factors. However, it is important to emphasize that observations of potential strengths, such as cautious driving or better lane control, are based on very few studies and should therefore be regarded as preliminary rather than generalizable findings.

Of particular importance is the finding that cognitive and brain functioning emerged as key predictors of driving ability. This demonstrates that deficits in driving competence are not merely behavioral but rooted in measurable neurobiological dysfunctions. These data support the need to integrate cognitive assessments and, where feasible, neurophysiological or neuroimaging data, into the evaluation of driving fitness in patients with schizophrenia.

Also, pharmacological treatment plays a dual role: some drugs improve functioning, but side effects (e.g., extrapyramidal symptoms, sedation) may undermine safety. Antipsychotic treatment should be judged not only by symptom control but also by its impact on real-world functioning. Functional assessments, such as neuropsychological batteries or simulator-based tests, can help clinicians evaluate how specific medications and their side effects influence driving safety. This underscores the need for individualized medication management, tailoring pharmacological choices to both clinical stability and functional outcomes.

From a clinical perspective, the evaluation of driving ability should be based on interdisciplinary collaboration among psychiatrists, neuropsychologists, occupational therapists, social workers and certified driving assessors, allowing for more comprehensive approaches. Within this framework, decisions regarding driving fitness should be individualized. Furthermore, targeted interventions such as psychoeducation and rehabilitation should be implemented. Finally, health systems and licensing authorities must acknowledge that blanket driving restrictions may be unjustified and ineffective if not based on individualized evaluations. These measures may improve safety and promote patient independence.

To strengthen the clinical perspective, greater emphasis should be placed on how driving assessments can be incorporated into personalized care plans. Systematic integration of driving evaluations may serve not only as an indicator of functional capacity but also as a practical tool for designing targeted interventions that foster autonomy and community participation. Moreover, regular monitoring of driving skills alongside cognitive and pharmacological parameters could support clinicians in making individualized decisions that balance safety with the promotion of independence.

In addition to the factors identified in the present review, broader clinical and policy aspects may also influence driving ability in schizophrenia. Driving is closely related to quality of life and social participation [11,19] while comorbidities such as epilepsy and dementia are strictly regulated with regard to license renewal due to their impact on cognition and safety [20,41]. Hepatic encephalopathy may impair driving capacity even at minimal grades [81] and substance or alcohol use disorders, which are highly prevalent in schizophrenia, further compromise driving performance [41]. Moreover, specialties such as forensic medicine, otorhinolaryngology, and ophthalmology, as well as general practitioners—who in several countries hold responsibility for certifying fitness to drive—play a pivotal role in the assessment process [82,83,84]. Although these aspects were not directly examined in the studies included in our review, they represent meaningful areas for future interdisciplinary research and clinical consideration.

Lastly, legal and policy issues are central to evaluating driving fitness in schizophrenia. Current frameworks often lack harmonization and rely on broad criteria, risking disproportionate restrictions. Recent evidence shows that policy alone is insufficient: a UK quality improvement project revealed major gaps in adherence to fitness-to-drive assessments despite formal guidelines [82], while a Canadian study highlighted clinicians’ uncertainty, limited resources and weak feedback from licensing authorities [84]. Evidence from the United States further indicates that relaxing renewal requirements for older drivers is associated with increased crash risk [85], reinforcing the need for rigorous procedures. Moreover, psychiatric populations face fragmented and inconsistent assessment practices, underlining the importance of standardized, multidimensional tools and professional training [83]. Collectively, these findings emphasize that effective policy must integrate cognitive and functional evaluation, ensure clinician support, and promote inter-sectoral collaboration to balance public safety with autonomy and social inclusion.

### 4.6. Strengths and Limitations

A notable strength of this systematic review is its focus on an underexplored research area, as the issue it addresses has received limited attention within the scientific community. Furthermore, this review emphasizes the importance of personalized treatment approaches to enhance and optimize driving performance in patients with schizophrenia, promoting their autonomy and community integration. Furthermore, rigorous scientific criteria were applied for the final selection of studies to ensure the scientific validity and reliability of the findings. Finally, the process strictly followed the PRISMA guidelines, ensuring high reporting quality. In addition, the quality of the included articles was assessed using the JBI tools, which provide a wide range of instruments for evaluating different study types.

Regarding limitations, it should be noted that only a small number of relevant studies were identified, as only six studies, all originating from Europe and Asia, met the inclusion criteria and were incorporated in the final analysis, which limited the robustness and breadth of the conclusions drawn. The inclusion of only six studies substantially reduces the strength of the evidence and calls for cautious interpretation of the findings. Also, the predominance of simulator-based assessments limits the ecological validity of the available evidence, as only one study employed real-world on-road testing. Another limitation is the exclusive inclusion of English-language studies, which may have led to the omission of relevant evidence published in other languages and therefore reduced the comprehensiveness of the review. Moreover, due to the high heterogeneity of study designs, outcome measures, and methodological approaches, a quantitative meta-analysis was not feasible. This absence of statistical pooling restricted our ability to quantify the strength of associations and instead necessitated a narrative synthesis.

Moreover, the inability to include gray literature or trial registries may have increased the risk of publication bias, as significant findings are more likely to be published while null results often go unreported [86,87]. Lastly, another limitation is that none of the included studies reported whether participants underwent standardized complementary investigations to exclude organic causes of psychosis. Consequently, we cannot completely rule out the possibility of diagnostic misclassification, such as pseudo-schizophrenia, organic schizophrenia, or secondary schizophrenia.

## 5. Conclusions

The present systematic review synthesized findings from only six original studies, highlighting that the current evidence base on driving performance in individuals with schizophrenia remains very limited. While the available data provide useful insights, they are constrained by several important gaps, such as low ecological validity, absence of interventional or rehabilitation studies and restricted geographical distribution of research. From this body of evidence, it emerged that driving ability of individuals with schizophrenia is shaped by a complex interaction of cognitive, clinical, experiential, and pharmacological factors. The findings indicate that while patients with schizophrenia often exhibit cognitive impairments and slower driving, they do not necessarily demonstrate riskier driving behavior in critical situations and may even show strengths in some respects. Nonetheless, these potential strengths have only been reported in very few studies and thus should be interpreted with caution and not generalized. Driving performance in this population is influenced not only by cognitive deficits, but also by factors such as driving experience and psychiatric history. Additionally, motor side effects from medication, especially extrapyramidal symptoms and the type of antipsychotic used, play a significant role in shaping driving ability. These results emphasize the multifactorial nature of driving competence in schizophrenia and the need for individualized assessments that consider both cognitive function and pharmacological factors.

A key finding of this systematic review is that the issue of driving ability in individuals with schizophrenia remains significantly underexplored. Although existing research is valuable, concise, and informative, there is a clear need for further expansion and strengthening of the scientific evidence base. More studies should be conducted under real-world conditions, as the current data reveal a substantial lack of ecologically valid assessments. Notably, only one of the studies included in this review was conducted in a real-life setting. It should be emphasized that the certainty of the available evidence on real-world driving performance is low. Therefore, current findings should be interpreted with caution, and future research with ecologically valid on-road assessments is essential to strengthen the evidence base.

There is also a clear research gap concerning the evaluation of therapeutic interventions and their effects on driving skills in patients with schizophrenia. Future research should therefore prioritize longitudinal study designs and interventional approaches, including cognitive remediation and rehabilitation programs, to directly evaluate personalized therapeutic approaches and their impact on driving ability in schizophrenia. Moreover, the present systematic review paves the way for more person-centered interventions which, if implemented in clinical practice, could offer meaningful benefits tailored to the individual needs and personal profile of each patient. A key implication of this review is the urgent need for the development and adoption of standardized protocols for assessing driving ability in individuals with schizophrenia, in order to ensure consistency, comparability and clinical applicability across studies and practice. Nevertheless, it is important to stress that these conclusions are drawn from only six studies, underscoring the preliminary nature of the evidence and the urgent need for more high-quality research in this field.

Lastly, future research should also address broader clinical and policy aspects, including comorbidities, substance use, and the role of interdisciplinary specialties and general practitioners in certifying driving ability, as these represent important dimensions not captured in the current evidence. Particular emphasis should be placed on longitudinal investigations, interventional trials, and ecologically valid on-road assessments, which will provide a more comprehensive and realistic understanding of driving performance in this population. Such studies, alongside the development of standardized and internationally applicable protocols, are essential for advancing both clinical decision-making and public safety.

## Figures and Tables

**Table 1:** Inclusion and exclusion criteria applied during the selection stages (screening and eligibility).

Inclusion Criteria	Exclusion Criteria
Individuals diagnosed with schizophrenia	Studies in animals
English language	Studies in minors, in healthy populations, in populations with other diseases
Full text research articles	Secondary analyses, reviews, guidelines, meeting summaries, comments, unpublished abstracts
Studies published within the last ten years, specifically between 2015 and 2025	

**Table 2:** Summary of JBI quality appraisal assessments- analytical cross-sectional studies: quality assessment [30].

	Q1. Were the Criteria for Inclusion in the Sample Clearly Defined?	Q2. Were the Study Subjects and the Setting Described in Detail?	Q3. Was the Exposure Measured in a Valid and Reliable Way?	Q4. Were Objective Standard Criteria Used for Measurement of the Condition?	Q5. Were Confounding Factors Identified?	Q6. Were Strategies to Deal with Confounding Factors Stated?	Q7. Were the Outcomes Measured in a Valid and Reliable Way?	Q8. Was Appropriate Statistical Analysis Used?
1. Okada et al., 2025 [31]	√	√	√	√	√	√	√	√
2. Biedermann et al., 2022 [14]	√	√	√	√	√	√	√	√
3. Noh et al., 2020 [32]	√	√	√	P	P	√	√	√
4. Okada et al., 2024 [33]	√	√	√	√	√	√	√	√
5. Steinert et al., 2015 [34]	√	√	√	√	√	√	√	√
6. Fuermaier et al., 2018 [35]	√	√	√	√	√	X	√	√
Overall appraisal	I	I	I	I	I	I	I	I

√: Yes, X: No, P: Partially, I: Include.

**Table 3:** Study origin, study type, patients’ and controls’ groups characteristics.

First Author, (Year)	Study Origin	Patients’ N (Male/Female)	Patients’ Age (Mean ± SD)	Control (Y/N)	Controls’ N (Male/Female)
1. Okada et al., 2025 [31]	Asia (Japan)	20 (12M-8F)	46.3 ± 11.1	Y	20 (12M-8F)
2. Biedermann et al., 2022 [14]	Europe (Germany)	50 (28M-22F)	43.1 ± 10.5	N	NA
3. Noh et al., 2020 [32]	Asia (South Korea)	102 (49M-53F)	45	N	NA
4. Okada et al., 2024 [33]	Asia (Japan)	42 (29M-13F)	42.7 ± 7.8	N	NA
5. Steinert et al., 2015 [34]	Europe (Germany)	150 (94M-56F)	45.2 ± 12.3	N	NA
6. Fuermaier et al., 2018 [35]	Europe (Germany)	31 (22M-9F)	29.9 ± 7.9	Y	31 (16M-15F)

Y: Yes, N: No, M: Male, F: Female.

**Table 4:** Overview of Neuropsychological and Neuroanatomical Findings Related to Driving Ability.

First Author, (Year)	Cognitive Deficits and Other Difficulties	Clinical and Neuropsychological Testing	Driving Assessment	Brain Scan and Findings	Main Findings
1. Okada et al., 2025 [31]	Sustained attention, visual processing speed, divided attention, and selective attention, Cognitive errors	PANSS, DIEPSS, TMT-A, CPT, WMS, ZMT, UFOV, SLOF	On-road	No	Patients drove more slowly and committed fewer speeding or distraction-related violations than controls. Driving violations were linked to cognitive deficits. Sudden braking was associated with extrapyramidal symptoms rather than road conditions.
2. Biedermann et al., 2022 [14]	Μild residual symptomatology, disorganization	PANSS, MSAS, Vienna Test System	Simulator	No	44% judged fit to drive, 20% partially fit, 36% unfit. Poorer fitness associated with older age, higher antipsychotic doses, and extrapyramidal symptoms.
3. Noh et al., 2020 [32]	Attention, Memory, Depth Perception, Executive Functioning	PANSS, CPAD	Simulator	No	Patients on aripiprazole or paliperidone performed better on driving-related cognitive tests (attention, memory, depth perception) than those on haloperidol or risperidone.
4. Okada et al., 2024 [33]	Processing Speed, Executive Function, Βrake Reaction Time, Memory, Disorganized	PANSS, TMT-A/B, WMS-R, ZMT	Simulator	Yes (fNIR) Reduced prefrontal cerebral blood flow	Reduced prefrontal activation and slower visual processing linked to delayed braking. Steering control influenced by visual memory, driving experience, and depressive symptoms. Limited planning and slower reactions increased collision risk.
5. Steinert et al., 2015 [34]	Cognitive dysfunction in attention, visuomotor coordination, processing speed	TMT-A, GAF	Self-reported questionnaire	No	64% held a driving license, but only 32% drove in the past year. License withdrawal (24.7%) linked to low functioning, repeated hospitalizations, and substance-related offenses.
6. Fuermaier et al., 2018 [35]	Divided Attention, Working Memory, Inhibition, Processing Speed, Planning, Hazard Perception	COGBAT, WAFS, WAFG, TMT, ToL, HPT, N-Back, Go/No-Go, VTS, MCVT-B	Simulator	No	Cognitive deficits in attention, processing speed, planning, and inhibition were evident. Patients did not show more crashes or delayed reactions than controls, but drove slower and caused hindrance during motorway merging.

Full names of the clinical and neuropsychological assessments used in the included studies. TMT **=** Trail Making Test, CPT **=** Continuous Performance Test, WMS **=** Wechsler Memory Scale, WMS-R = Wechsler Memory Scale-Revised, ZMT = Zoo Map Test, UFOV = Useful Field of View, SLOF = Specific Level of Functioning Scale, PANSS = Positive and Negative Syndrome Scale, DIEPSS = Drug-Induced Extrapyramidal Symptoms Scale, MSAS = Modified Simpson Angus Scale, CPAD = Cognitive Perceptual Assessment for Driving, GAF = Global Assessment of Functioning, COGBAT = Cognitive Basic Assessment Battery, WAFS/WAFG = Selective/Divided Attention Tests, ToL = Tower of London, HPT = Hazard Perception Test, VTS = Vienna Test System, MCVT-B = Multiple Choice Vocabulary Test B, EPS = Extrapyramidal Symptoms.

**Table 5:** Cognitive domains associated with driving performance in people with schizophrenia.

Cognitive Domain	Main Associations with Driving
Attention (sustained, selective, divided)	Related to driving violations, delayed responses, and reduced situational awareness
Processing Speed	Associated with slower reactions and difficulties in merging or adapting to traffic flow
Executive Functions (planning, inhibition, decision-making)	Linked to impaired lane control, risk anticipation, and driving errors
Memory (working/visual)	Influenced steering control, adaptation to unexpected events
Visuospatial Skills	Associated with depth perception and distance judgment while driving
Hazard Perception	Connected to increased collision risk and reduced hazard anticipation

## Data Availability

No new data were created or analyzed in this study. Data sharing is not applicable to this article.

## Supplementary Materials

The following supporting information can be downloaded at: https://www.mdpi.com/article/10.3390/brainsci15101094/s1. Supplementary File S1 (PRISMA 2020 checklist) can be downloaded from the electronic submission system of the journal.
